# Differences Between the Oral Health of People Aged 50 and 70 Years – An Exploratory Cohort Study

**DOI:** 10.3290/j.ohpd.a43363

**Published:** 2020-07-04

**Authors:** Anna-Luisa Klotz, Sabine Katharina Grill, Alexander Jochen Hassel, Peter Rammelsberg, Andreas Zenthöfer

**Affiliations:** a Dentist, Department of Prosthetic Dentistry, Dental School, University of Heidelberg, Heidelberg, Germany. Managed the literature searches and wrote the manuscript; data analysis, drafting, critically revising and approval of the final paper.; b Dentist, Department of Prosthetic Dentistry, Dental School, University of Heidelberg, Heidelberg, Germany. Data generation, interpretation and analysis; drafting, critically revising and approval of the final paper.; c Research Associate, Department of Prosthetic Dentistry, Dental School, University of Heidelberg, Heidelberg, Germany. Designed the study, wrote the protocol and undertook the statistical and data analysis; drafting, critically revising and approval of the final paper.; d Chair, Department of Prosthetic Dentistry, Dental School, University of Heidelberg, Heidelberg, Germany. Design of the study and data interpretation and analysis; drafting, critically revising and approval of the final paper.; e Associate Professor, Department of Prosthetic Dentistry, Dental School, University of Heidelberg, Heidelberg, Germany. Designed the study, wrote the protocol and undertook the statistical and data analysis; managed the literature searches and wrote the manuscript; drafting, critically revising and approval of the final paper.

**Keywords:** caries prevalence, periodontitis, oral health, oral hygiene, elderly people

## Abstract

**Purpose:**

To assess the extent of differences between the oral health of people aged 50 and 70 years in a community-based setting.

**Materials and Methods:**

This research is part of the Interdisciplinary Study on Adult Development (ILSE). All participants lived in the city of Heidelberg, Germany. For the dental study, 194 participants born 1930–1932 (n = 88) or 1950–1952 (n = 106) underwent a comprehensive dental examination. For each participant the number of teeth present, the number of decayed, missing, and filled tooth surfaces (DMF-S), the Plaque Index (PI), the Gingiva Index (GI) and the Community Index of Periodontal Treatment Needs (CPITN) were determined. Depending on the structure of the data, differences between the birth cohorts were calculated by use of t tests or chi-squared tests. Multivariate analysis was also performed to assess possible effects of gender and birth cohort.

**Results:**

Oral health conditions were significantly worse among septuagenarians than among quinquagenarians. Besides poorer oral hygiene, as measured by use of PI and GI (p <0.001), periodontal conditions were worse for septuagenarians (p <0.001), who also had fewer natural teeth (p <0.002); the number of carious lesions was similar in the cohorts (p >0.05). These results were confirmed by multivariate analysis and seem to be mostly gender independent.

**Conclusions:**

Oral hygiene and health is poor for quinquagenarians and septuagenarians, with more problems associated with greater age but not with gender. Longitudinal studies are necessary to evaluate the intraindividual development of changes of oral health during ageing.

Demographic changes are leading to a greater proportion of elderly people worldwide. This ageing population is a challenge to dental care, because elderly people face a variety of oral problems and their treatment needs differ from those of younger people.^29^ Oral disease has several causes, however, including lifestyle, general health problems and sociodemographic and psychological aspects, which accumulate with ageing. It is also difficult to assess dental health services.^21^ Paradoxically, routine utilisation of dental check-ups decreases with age, resulting in later recognition of diseases, more severe disease, and, therefore, tooth loss.^6,7^

The major cause of such oral diseases as caries and periodontitis is inadequate oral hygiene.^4,6,25^ In many Western countries, however, notable improvement of oral health, even into old age, has been associated with more dedicated preventive measures and improved conservative treatment.^1,17^ Caries and periodontitis are, however, still serious problems throughout the world, primarily among the elderly. For caries in Germany, for example, the DMF tooth index for people aged 35–44 and 65–74 years is 14.5 and 22.1, respectively.^17^ In a British study mean (SD) DMF-S for participants 60 years and above was 85 (52.3), and greater age was linked to worse DMF.^3^ The association between age, caries and tooth loss has been confirmed by another study.^10^ Periodontal disease is also a serious problem which is highly prevalent in adults and elderly people.^17^

In countries in which mild and moderate periodontitis affected most adults, studies have reported a prevalence of severe periodontitis of between 5 and 50%, depending on country.^13–15^ An association between age and the prevalence and severity of the disease has also been reported.^8,9^ Caries and periodontitis, and their clinical manifestations, are the most common reasons for tooth loss. The prevalence of edentulism is increasing in many developed countries – substantial tooth loss with increasing age is still a reality.^19,29^ This leads to reduced chewing ability, poorer oral health-related quality of life, and changes in nutrition.^5,18^ Besides the more or less direct effects on oral health (plaque and periodontitis), dental pathogens have also been shown to be associated with such general health problems as pneumonia, diabetes mellitus and cardiovascular disease.^12,22,24^

Except for institutional studies, no information is available about the oral health of quinquagenarians and septuagenarians. The objective of this study was, therefore, to investigate the oral hygiene (Plaque Index, Gingiva Index) and health (DMF-S, periodontal disease) of representative community-based samples of people aged 50 or 70 years.

## MATERIALS AND METHODS

### Study Population

This study is part of the Interdisciplinary Study on Adult Development (ILSE), an interdisciplinary study of the psychological and medical characteristics of two birth cohorts born 1930–1932 (older cohort; OC) or 1950–1952 (younger cohort; YC).^23^ In the 1990s, 500 participants living in Heidelberg, Germany were randomly selected by a citizen administration office; they were required to be representative with regard to gender distribution (first group of measurements). The study was complemented by a dental study (third group of measurements). The local review board approved the amendment to enable study of dental aspects (#181/2005). After acquisition of detailed oral and written information for 230 feasible ILSE participants, 88 and 106 people were assigned to OC and YC, respectively (n = 194) and subjected to complete dental examinations. The only inclusion criterion was signed informed consent.

### Collection of Target Variables

The dental examinations were performed by three dentists trained at the Department of Prosthodontics of the University of Heidelberg. Mouth mirrors, and dental and periodontal probes were used for the examinations. For each participant, dental status (number of teeth present and number of prosthetic restorations) was determined. Oral hygiene was evaluated by use of the Gingiva Index (GI) and the Plaque Index (PI).^26^ Both indices are graded on a four-point scale (0 = no plaque and no bleeding to 3 = substantial plaque accumulation and severe bleeding). PI and GI were recorded at two sites for each tooth. For estimation of periodontal conditions, the Community Index of Periodontal Treatment Needs (CPITN) was used.^29^ CPITN can range from 0 (no periodontal finding) to 4 (severe periodontitis).^2^ Decayed (D), missing (M), and filled (F) tooth surfaces were recorded by use of the DMF-S index.^28^ Third molars were excluded from calculations and posterior teeth were evaluated at five sites whereas anterior teeth were evaluated at four sites. Scores for D, M and F could, therefore, each range from 0 to 128.^29^ Gender and cohort membership were also noted on the case record forms.

### Statistical Evaluation

Statistical analysis was performed by use of SPSS version 23.0 (IBM, New York, USA). Statistical significance was observed at α <0.05.

Descriptive statistics (means and standard deviations or frequencies and percentages) were plotted for the target variables for both cohorts. Differences between OC and YC were analysed by use of t tests (interval) or chi-squared tests (binominal), depending on data structure. A linear regression model was also calculated for each dependent dental target variable, with cohort membership and gender as confounders.

## RESULTS

### Study Population

One-hundred and ninety-six (196) participants, from 230 initially feasible participants were considered for statistical analysis. Thirty-six participants had to be excluded because of missing target variables. One-hundred and six participants were assigned to YC and 88 to OC. Mean (SD) age of the sample was 63.5 (9.5). Gender distribution was balanced (49.5% female).

### Oral Hygiene and Health

The mean (SD) number of own teeth for the sample was 20.4 (8.8), with 24.3 (5.9) and 15.7 (9.4) for YC and OC, respectively. Only 5.7% of the participants were edentulous. Most (64%) of the participants had fixed dental prostheses in both jaws; 22% and 13% wore removable dental prostheses and complete dentures, respectively, in at least one jaw.

Mean (SD) DMF-S for decayed surfaces was 0.9 (2.1); for filled and missing surfaces, the mean was nearly 40. Mean (SD) PI and GI were 0.7 (0.6) and 0.4 (0.4), respectively. Mean (SD) CPITN for the sample was 2.5 (0.8). Statistically significant (p <0.002) differences between the cohorts were detected for all the dental target variables except decayed surfaces, for which, however, a substantial trend toward more caries in OC was observed (p = 0.081). Detailed measures of central tendency and variation, and results from bivariate analysis are given in Table 1.

**Table 1:** Participants’ characteristics and bivariate comparison of target variables for the younger (YC) and older (OC) cohorts

	Total sample(n = 194 )	YC (n = 106)	OC (n = 88)	p value
Gender, # (%)				
Male	98 (50.5%)	52 (49.1%)	46 (52.3%)	= 0.429
Female	96 (49.5%)	54 (50.9%)	42 (47.7%)	
Dental status, # (%)				
With natural teeth	183 (94.3%)	105 (99.1%)	78 (88.6%)	**<0.002**
Edentulous	11 (5.7%)	1 (0.9%)	10 (11.4%)	
Decayed surfaces, mean (SD)	0.9 (2.1)	0.7 (2.0)	1.2 (2.2)	= 0.081
Missing surfaces, mean (SD)	39.8 (37.9)	22.6 (25.2)	60.5 (40.4)	**<0.001**
Filled surfaces, mean (SD)	38.5 (22.1)	46.0 (20.4)	29.4 (20.8)	**<0.001**
Plaque Index, mean (SD)	0.7 (0.6) *	0.4 (0.4) **	1.2 (0.5) ***	**<0.001**
Gingiva Index, mean (SD)	0.4 (0.4) *	0.3 (0.4) **	0.5 (0.4) ***	**<0.001**
CPIN, mean (SD)	2.5 (0.8) *	2.3 (0.9) **	2.8 (0.5) ***	**<0.001**

Statistically significant p values are marked in bold. * n = 183; ** n = 105; *** n = 78; # = number of participants.

### Multivariate Analysis

The bivariate effect of cohort membership was confirmed by multivariate analysis, with gender as confounder. Although gender did not significantly affect the values of the target variables, a trend (p <0.100) towards different conditions was observed for decayed and filled tooth surfaces, which were more prevalent among women, and for PI, which was worse for men. Detailed results are presented in Table 2.

**Table 2:** Linear regression analysis for the dependent dental target variables, with cohort and gender as confounders

Confounder	95% Confidence interval			
	Regression	Lower border	Upper limit	p value
Decayed (n = 194)				
Female	0.16	−0.43	0.075	0.059
Older cohort	0.53	−0.06	1.12	**0.076**
Missing (n = 194)				
Female	0.01	−9.36	9.37	0.999
Older cohort	37.83	28.43	47.24	**0.001**
Filled (n = 194)				
Female	5.1	−6.67	10.94	0.082
Older cohort	−16.47	−22.30	−10.65	**0.001**
Plaque Index (n = 183)				
Female	−0.11	−0.23	0.02	0.096
Older cohort	0.86	0.74	0.99	**0.001**
Gingiva Index (n = 183)				
Female	−0.06	−0.17	0.06	0.322
Older cohort	0.28	0.16	0.40	**0.001**
CPITN (n = 183)				
Female	−0.19	−0.42	0.04	0.102
Older cohort	0.45	0.22	0.68	**0.001**

Statistically significant p values are marked in bold.

## DISCUSSION

This study observed statistically significant differences between several aspects of oral health among quinquagenarians and septuagenarians. Oral hygiene and periodontal conditions seemed worse among the older participants, whereas the prevalence of caries was not. Plaque accumulation and gingival bleeding were significantly greater in the older cohort. The association found between age and oral hygiene agrees with available literature and was not surprising. Between the age of 50 and 70 years, the Plaque Index triples from more or less acceptable oral hygiene (mean PI 0.4) to moderate but widespread plaque accumulation (mean PI 1.2), indicating a relevant shift in this age range. Oral hygiene depends – among other variables – on motor ability, which can be lower for elderly people.^21^ The relevance of oral hygiene and health can, moreover, decrease during ageing because of the effect of other, more severe, systemic diseases.

In this study, oral hygiene was evaluated by use of the Plaque and Gingiva Indices. Both are established indices used in clinical routine and in epidemiological surveys. One might question why two instruments were used for estimation. The Plaque Index, however, gives a snapshot, only, of oral health; complementation by use of the Gingiva Index enables estimation of longer-term oral hygiene.^26^ This is relevant, because some participants underwent other examinations before and after the dental examination, in the context of this study, and eating and brushing behaviour on the day of the study was not assessed. Other indices, for example the Plaque Control Record are more comprehensive, because plaque is tinted by use of a plaque indicator.^20^ Use without subsequent professional tooth cleaning is not well accepted by patients, however. It is also worthy of note that periodontal conditions were found to be worse among septuagenarians. This is not surprising, because gingivitis and periodontitis are a long-term result of poor oral hygiene. In this study the CPITN was used for estimation of periodontal treatment need to ensure comparability with other studies. The CPITN is a popular index recommended by the WHO for measurement of periodontal treatment needs in dental epidemiology.^29^ The prevalence of periodontitis increases with age, peaks at an approximate age of 40 years, and remains stable at greater ages.^13^

The observation that periodontal destruction increases with age is in accordance with the results of the present study. In this study, participants in both groups had severe periodontal problems, but the consequences were distinct in the OC. This could be explained by a notable reduction of dental visits after the age of 50.^7^ The increased periodontal destruction could also be explained by greater prevalence of systemic diseases and intake of medication, which can affect oral disease.^12,24^ Although the effect of gender has been discussed elsewhere,^8^ this study found no differences between gender, in accordance with Kassebaum et al (2014).^13^ Closer investigation revealed the mean prevalence of moderate periodontitis was 43.4% between the ages of 65 and 75 years and 45.7% for 75–100 year olds, whereas the incidence of severe periodontitis was 19.8% and 44.3% among the YC and OC in Germany.^15^

Severe periodontal disease – as measured by PSI code 4 in at least one sextant – was observed for nearly half of our study sample. This was higher than that found among participants of similar ages in other studies in Europe, and supports the findings of König et al (2010), who found a greater prevalence of periodontitis in Germany and the United Kingdom than in Spain, Sweden and Switzerland.^14^

The other important aspect analysed in our study was dental caries. The incidence of dental caries was high along our sample, especially among the older cohort, which confirms the results of the German National survey on oral health.^17^ The DMF-S is the WHO-recommended method for measurement of the incidence of caries in dental epidemiology.^28^ The DMF-S (84.8) reported by Al-Haboubi et al (2010)^3^ for a London population of 60 years and older was higher than that in our YC (68.6) but lower than that in our OC (91.1); this supports the hypothesis of severe oral changes during this age range. Kassebaum et al (2014) reported the mean prevalence of global untreated caries (D-T) was 35%.^13^ This is higher than the result in our study, in which the prevalence was 26.8%. Despite a clear trend to more carious tooth surfaces in the older cohort, the difference was not statistically significant. This could be because tooth loss increases with increasing age, which might have diluted possible differences. This assumption is supported by differences between filled tooth surfaces in the cohorts; significantly more filled surfaces were observed in the YC. Several studies have reported an increased risk of tooth loss with ageing.^4,6,25^ This is as expected, because caries and periodontal disease – highly prevalent among the elderly – lead to non-preservability of teeth. The number of natural teeth among our study population was slightly higher than, but comparable with, that observed in other studies.^19^ The prevalence of edentulism was, however, lower than that found in previous studies (All: 5.7%; OC: 11.4%). In Brazil, for example, edentulism among the elderly was approximately 40%; in Manhattan (USA) it was 19.5%.^11,19^ This supports the idea that our sample was healthier than in other countries. Because tooth loss is associated with reduced chewing function and, therefore, a decrease in oral health-related quality of life,^5,18^ it is important to recognise oral diseases at an early stage. Because the results of this study suggest deterioration of oral health between the ages of 50 and 70, prevention and adequate conservative treatment for those in this age range seem especially important. This could be enabled by establishing prophylaxis strategies for the elderly.

### Strengths and Weaknesses of the Study

Drop-out of approximately 40% in this study suggests caution in interpretation and generalisation of the results. Participants were, perhaps, more interested in their oral health than were non-responders. It is also possible that some of the participants suspected acute dental problems and participated for this reason. The sample is, however, a subpopulation from a large, representative interdisciplinary study and all feasible participants who wanted to participate were included.

## CONCLUSION

In conclusion, this study revealed statistically significant differences between the oral health of people aged 50 and 70 years. Poor oral hygiene, compromised periodontal conditions and tooth loss were associated with old age whereas caries was comparable in both age cohorts. Frequent dental visits between the ages of 50 and 70 years would be desirable to prevent deterioration of oral conditions. Longitudinal studies are necessary to confirm these results and to investigate intraindividual developments.

### Ethical Approval

All procedures performed in this study involving human participants were in accordance with the ethical standards of the institutional and/or national committee and with the 1964 Helsinki declaration and its later amendments or comparable ethical standards.
